# The Use of Vasopressor Agents in Free Tissue Transfer for Head and Neck Reconstruction: Current Trends and Review of the Literature

**DOI:** 10.3389/fphar.2020.01248

**Published:** 2020-08-28

**Authors:** Akash N. Naik, Taylor Freeman, Michael M. Li, Scarlett Marshall, Akina Tamaki, Enver Ozer, Amit Agrawal, Stephen Y. Kang, Matthew O. Old, Nolan B. Seim

**Affiliations:** ^1^Department of Otolaryngology-Head and Neck Surgery, The Ohio State University Wexner Medical Center, Columbus, OH, United States; ^2^College of Medicine, The Ohio State University, Columbus, OH, United States; ^3^Department of Anesthesia, The Ohio State University Wexner Medical Center, Columbus, OH, United States; ^4^Department of Otolaryngology-Head and Neck Surgery, University Hospitals Cleveland Medical Center, Cleveland, OH, United States

**Keywords:** vasopressors, anesthesia management, microvascular surgery, head and neck reconstruction, free tissue transfer

## Abstract

**Background/Objectives:**

Microvascular free tissue transfer has become essential to head and neck reconstruction and recent advancements in microvascular surgery have led to excellent surgical outcomes. However, there continues to be controversy and a stigma associated with the use of perioperative intravenous vasopressor agents among both surgeons and anesthesiologists. Due to concern for vasoconstriction of peripheral vasculature flowing to the denervated tissue flap, there remains concerns about potential thrombosis, decreased tissue perfusion and ultimately flap failure. This topic becomes even more important as vasopressors play an essential role in new Extended Recovery After Surgery (ERAS) protocols being put in place to optimize postoperative recovery for patients. The purpose of this study was to comprehensively review the role and safety as well as discuss current trends with intraoperative vasopressor agents in free tissue transfer for head and neck reconstruction.

**Methods:**

A scoping literature review was conducted of all studies that examined the use of vasopressor agents during head and neck free flap tissue transfer. Primary and secondary outcomes included free flap survival, arterial thrombosis, venous congestion, need for revision surgery, and other postoperative complications.

**Results:**

One prospective and nine retrospective studies were identified. Phenylephrine and ephedrine were the most common vasopressors reported; the rate of vasopressor use ranged from 53% to 85% and administration methods included both bolus and infusion. The included studies did not show any significant association between the use of vasopressors and free flap failure, pedicle thrombosis, or other flap complications.

**Conclusion:**

The administration of vasopressors during microvascular free tissue transfer for head and neck reconstruction does not seem to be associated with increased flap failure rates or other postoperative morbidities. Moreover, vasopressors may provide overall improved hemodynamic stability and help to limit overall fluid administration and subsequent postoperative complications. Additional prospective investigation is warranted to further elucidate and establish evidence-based recommendations regarding the type, timing, and dose of vasopressors to further enhance free flap survival and patient outcomes.

## Introduction

Microvascular free tissue transfer (MFTT) has become an essential component of head and neck reconstruction (Monroe et al., 2010). While advancements have led to excellent surgical outcomes with MFTT success rates routinely exceeding 95% (Densky et al., 2019), there remains controversy regarding the use of intraoperative intravenous vasopressor agents among both surgeons and anesthesiologists during these procedures (Harris et al., 2012; Chan et al., 2013; Ibrahim et al., 2014; Swanson et al., 2016; Wax and Azzi, 2018). Due to intraoperative hypotension, it is commonplace for teams to have ongoing discussions during these cases to select intraoperative fluid administration or vasopressor therapy medication and dosing. Theoretically, vasoactive agents have been feared to cause vasoconstriction, thereby potentially increasing the risk of thrombosis and flap failure (Cordeiro et al., 1997; Harris et al., 2012; Ibrahim et al., 2014). This was supported, early on, by animal models suggesting that phenylephrine decreased flow through the flap pedicle in musculocutaneous island flaps (Cordeiro et al., 1997). Thus, vasopressors have often been historically and anecdotally associated with potential postoperative complications with 70% of surveyed surgeons prohibiting their use during microvascular surgery in a previous study (Motakef et al., 2015; Chang et al., 2017). More recently, however, studies have challenged this paradigm and demonstrated an increased prevalence of intraoperative vasopressor use in free flap reconstruction without a significant impact on MFTT outcomes (Monroe et al., 2011; Harris et al., 2012; Chan et al., 2013; Swanson et al., 2016; Fang et al., 2018; Goh et al., 2019).

While data and opinions regarding vasopressor use in MFTT are evolving, there is a lack of evidence-based guidelines that draw upon prospective studies. As a result, there exists a broad spectrum of practice regarding the administration and type of vasopressors used based on an institutional and personal basis. Moreover, this topic is especially relevant as vasopressor agents play an essential role in newly implemented Extended Recovery After Surgery (ERAS) protocols, which universally limit intraoperative fluid administration with goals to improve recovery time and outcomes. The purpose of this study is to comprehensively review the role, safety, and current trends of intraoperative vasopressor agents in MFTT for head and neck reconstruction.

## Methods

### Literature Search Strategy

A comprehensive scoping literature review was conducted through the PubMed-NCBI, Google Scholar, and Scopus. The final search was completed in February 2020. The search encompassed terms “vasopressor” OR “vasoconstrictive agents” AND “microvascular free tissue transfer” OR “free flap” AND “head and neck reconstruction”. An English-language filter was applied. Each database was searched from inception date until February 2020. The reference lists of all obtained articles were examined for additional studies meeting inclusion criteria. Two authors (AN and TF) independently conducted the searches. The senior author was consulted for inclusion of seminal articles on the topic (NS). The resulting studies were reviewed first through titles and abstracts followed by full manuscript review of abstracts meeting inclusion criteria upon initial review.

### Exclusion Criteria and Outcomes

Studies not in English and those examining MFTT results outside of the head and neck were excluded. Additionally, case reports and small case series were excluded. For studies that included overlapping series of patients, the most recent study with the largest number of patients was selected. The primary variable examined was the type of vasopressor agent and secondary variables included method and timing of administration. The primary outcome was the rate of free flap failure. Secondary outcomes included arterial thrombosis, venous congestion, and other postoperative complications including revision surgery, intraoperative re-anastomosis, wound infections, dehiscence, or hematoma.

### Review of Vasopressor Use in Head and Neck MFTT

The search returned 247 articles. After applying exclusion criteria and reviewing abstracts, eight studies (one prospective observational and seven retrospective) were identified for inclusion. The results of these studies are highlighted in **Table 1**.

**Table 1:** Flap Outcomes and Postoperative Complications Among Studies Examining the Effect of Intraoperative Vasopressors in Head and Neck Reconstruction.

Reference	Type of study	Free flap (%)	Use of intraoperative vasopressor, n (%)	No use of intraoperative vasopressor, n (%)	Type of vasopressor	Flap failure	Postoperative complications
Fang et al. (2018)	R	NR	2637 (88.4%)	346 (11.6%)	CaCl (77%) Eph (45%) Phe (21%)^#^	No significant difference in free flap failure rates. VP: 39/2637 (1.48%) vs Non-VP: 6/346 (1.73%), p=0.715	Pedicle compromise: -Intra-operative: VP: 8/2637 (0.30%) vs non-VP: 1/346 (0.29%) p=0.965 -Postoperative: VP: 76/2637 (2.88%) vs non-VP: 16/346 (4.62%), p=0.081 Arterial compromise: VP: 38/2637 (1.44%) vs non-VP: 10/346 (2.89%), *p=0.048* Venous congestion: VP: 59/2637 (2.23%) vs non-VP: 12/346 (3.47%), p= 0.161
Farquhar et al. (2018)	R	ALT (25.7%) RF (23.5%) Fibula (24.1%) Pectoralis (4.12%) Scapular tip (10.6%) LD (2.94%) RA (6.47%) Serra A (1.17%) Scapula (0.59%)	84 (54.5%)	70 (45.5%)	NR	No significant difference in free flap failure rates. VP: 11/84 (13.1%) vs Non-VP: 7/70 (10.0%), p=0.537	Major complications:^$^ VP: 37/84 (44.0%) vs non-VP: 30/70 (42.9%), p=0.882
Chang et al. (2017)	R	ALT (46.6%) RF (22.1%)) Fibula (15.3%)) VL (2.7%) LD (2.7%)	278 (66.3%)	141 (33.7%)	Eph (44.5%) Phe (48.4%) Eph + Phe (33.3%)	Total flap loss: 12/419 (2.86%) *No direct comparison between VP and no VP failure rate	Arterial complication, VP: 22/278 (7.9%) vs non-VP: 2/141 (1.42%), *p = 0.002* OR = 6 (1.26-28.4), *p = 0.024*
Rose et al. (2016)	R	RF (70.7%) Fibula (17.1%) ALT (4.07%) Jejunum (4.07%) RA (2.44%) DCIA (1.63%)	93 (75.6%)	30 (24.4%)	NE (49.5%) MTM (44.1%) NE + MTM (6.45%)	VP: 3/93 (3.23%) Non-VP: 1/30 (3.33%)	Cases requiring salvage procedure to sustain viability: VP: 8/93 (6.50%) non-VP: 3/30 (10.0%)
Chan et al. (2013)	R	Jejunum (100%)	81 (73.6%)	29 (26.4%)	Eph (42.7%) Phe (14.5%) Eph + Phe (42.8%)	2/2 AT: VP: 1/81 (1.23%) vs Non-VP: 2/29 (6.9%), p = 0.08 2/2 VT: VP: 1/81 (1.23%) vs Non-VP: 2/29 (6.9%), p = 0.08	Late stricture formation: VP: 2/81 (2.47%) vs non-VP: 1/29 (3.4%), p = 0.12 Intra-op anastomosis revision due to AT: VP 1/81 (1.23%) vs non-VP, 2/29 (6.9%), p = 0.08
Harris et al. (2012)	R	RF (32.3%) ALT (28.3%) Scapula (13.9%) Fibula (12.3%) LD (6.2%)	320 (66%)	165 (34%)	Phe (34.7%%) Eph (25.6%) Phe + Eph (37.2%) Other (2.50%)	No significant difference in free flap failure rates. VP: 8/320 (2.5%) vs Non-VP: 3/165(1.81%), p = 0.76	VP: 18/320 (5.6%) vs vs non-VP: 8/165 (4.8%), p = 0.72^!^ Timing of intra-op VP administration not significantly associated with adverse flap outcomes (p = 0.39)
Monroe et al. (2011)	P	RF (43.2%) ALT (16.6%) Fibula (15.4%) LD (10.1%) RA (8.28%) Jejunum (3.55%) Ulnar (1.77%) Scapula (1.18%)	90 (53.3%)	79 (46.7%)	Phe (63.0%) Eph (52.0%)	VP: 4/90 (4.44%) vs Non-VP: 2/79 (2.53%) 90% CI: -1.4 to 5.2	Total flap complications: VP: 34/90 (37.8%) vs non-VP: 34/79 (43.0%), p = 0.48 Medical complications: VP: 5/90 (5.56%) vs non-VP: 9/79 (11.4%), p= 0.17 Hematoma: VP: 8/90 (8.89%) vs Non-VP: 7/79 (8.86%), p = 0.99 Wound dehiscence: VP: 9/90 (10.0%) vs Non-VP: 9/79 (11.4%), p = 0.77 Infection: VP: 6/90 (6.67%) vs Non-VP: 6/79 (7.59%), p = 0.81 Anastomotic revision: VP: 3/90 (3.33%) vs non-VP: 2/79 (2.53%), p = 0.76 Other unspecified complications: VP: 7/90 (7.78%) vs Non-VP: 5/79 (6.33%), p= 0.76
Monroe et al. (2010)	R	RF (38.5%) RF w/bone (7.10%) Fibula (13.6%) ALT (12.4%) RA (10.1%) LD (7.10%) Ulnar (4.14%) Scapula (4.14%) Jejunum (2.96%)	139 (86.9%)	30 (13.1%)	Phe (33.0%) Eph (15.0%) Phe + Eph (51.0%) Dopa (1.00%)	No significant difference in free flap failure rates. VP: 4/139 (2.9%) vs Non-VP: 2/30 (6.7%), p= 0.29	Total flap complications: VP: 40/139 (28.7%) vs non-VP: 9/30 (30.0%), p = 1.00 Infection: VP: 9/139 (6.47%) vs non-VP: 1/30 (3.33%), p = 1.00 Dehiscence of fistula: VP: 10/139 (7.19%) vs non-VP: 2/30 (6.67%), p = 1.00 Hematoma: VP: 9/139 (6.45%) vs non-VP: 2/30 (6.67%), p = 1.00 Partial Loss: VP: 5/139 (3.6%) vs non-VP: 1/30 (3.33%), p = 1.00 Pedicle thrombosis: VP: 11/139 (7.91%) vs non-VP: 5/30 (1.67%), p = 0.17

R, retrospective study; NR - not recorded; CaCl, calcium chloride; Eph, ephedrine; Phe, Phenylephrine; VP, vasopressor; ALT, anterolateral thigh; RF, radial forearm; LD, latissimus dorsi; RA, rectus abdominis; Serr A, serratus anterior; VL, vastus lateralis; HR, adjusted Hazard ratio for logistic regression; DCIA, deep circumflex iliac artery; NE, norepinephrine; MTM, metaraminol; AT, arterial thrombosis; 2/2, secondary to; P -prospective observational; CI, confidence interval; Dopa, Dopamine.

#- Did not specify type of vasopressor used for head and neck reconstructions.

$- included reoperation, fistula, myocardial infarction, emergent tracheostomy, flap death, serious infection, and pulmonary embolus in the 30-day postoperative period.

! - complications not specified.

#### Prevalence and Types of Vasopressors

The most commonly used vasoconstrictive agents in the selected studies were ephedrine and phenylephrine, used alone or in combination (**Table 1**). The rate of intraoperative vasopressor use in the selected studies ranged from 53.3% to 88.4% and administration methods included both intravenous bolus and continuous infusion (Monroe et al., 2010; Monroe et al., 2011; Harris et al., 2012; Chan et al., 2013; Rose et al., 2016; Chang et al., 2017; Fang et al., 2018; Farquhar et al., 2018).

#### Free Flap Failure Rates and Postoperative Complications

Across the included studies, flap failure rate ranged from 1.48% to 13.1% with no study identifying a statistically significant difference between vasopressor and non-vasopressor groups. In the largest study, Fang et al. identified 2,983 patients who underwent head and neck MFTT and found that intraoperative vasopressor use was not associated with an increase in free flap failure rates (1.48% vs 1.73%, p = 0.72) (Fang et al., 2018).

Alternatively, in a study of 110 free jejunal flaps, Chan et al. observed no significant relationship between the use of intraoperative vasopressors and the need for intraoperative re-anastomosis, free flap failure rate, or long-term stricture rate (Chan et al., 2013). Similarly, Harris et al. also showed no statistically significant difference between the vasopressor and non-vasopressor group with regards to complete flap failure (2.5% vs 1.8%, p = 0.76) or major flap complication rate, defined as the total proportion of flaps that failed or required revision surgery (5.6% vs 4.8%, p = 0.72) (Harris et al., 2012).

In contrast, Chang et al. observed that the use of vasopressors was an independent risk factor for arterial complications (odds ratio (OR) = 6; p = 0.02) (Chang et al., 2017). Despite a three-fold higher risk for emergent return to the OR and additional major surgical complications, patients with intraoperative arterial complications did not have higher rates of free flap failure. Thus, it appears most free flaps were salvaged in this study when arterial issues arose. Unfortunately, there was no direct comparison for free flap failure rates between the vasopressor group and non-vasopressor group (Chang et al., 2017).

### Timing of Administration and Dosage

Chan et al. also demonstrated no relationship between the timing of vasopressor administration (i.e. prior to, during, or after free flap harvesting) and postoperative failure rates (Chan et al., 2013). An additional study concluded that pedicle compromise and flap failure rates were not associated with timing of intraoperative vasopressor use (p = 0.106 and p = 0.162, respectively) (Fang et al., 2018). Harris et al. showed no significant association between adverse flap outcomes and the timing of intraoperative vasopressors (first 3 h of the case, middle of the case, and last 3 h of the case; p = 0.39) or the cumulative dosage with either phenylephrine or ephedrine (p = 0.43 and p = 0.37, respectively) (Harris et al., 2012). In a prospective observational study, Monroe et al. observed no association between the total vasopressor dose administered and flap failure rates (Monroe et al., 2011).

## Discussion

The use of intraoperative vasopressors has long been debated among microvascular surgeons due to concerns for free flap failure and associated postoperative complications. Due to the lack of prospective data and heterogeneity of previous studies that include various defect sites and reconstruction methods, there is a lack of consensus on the use of intraoperative vasopressors.

### Theoretical Risk of Vasopressors

This controversy likely stems from the conventional theory that vasoactive drugs may result in vasospasm, decreased flap perfusion, and subsequent flap failure in a denervated flap (Godden et al., 2000; Harris et al., 2012; Goh et al., 2019). Of note, these postulations are primarily based on experimental animal models with conflicting results (**Table 2**) (Banic et al., 1997; Cordeiro et al., 1997; Massey and Surgery, 2007; Lecoq et al., 2008; Scholz et al., 2009; Eley et al., 2013).

**Table 2:** Summary of Commonly Used Vasopressors in Microvascular Surgery.

Vasopressor	Manuscript including agent	Description of agent	Pharmacologic target	Physiologic effects	Potential adverse effects	Flap effects based on experimental animal and human studies
Phenylephrine (Neo-Synephrine)	Chan et al. Chang et al. Fang et al. Harris et al. Monroe et al.	Synthetic non-catecholamine	Strong α-1 adrenergic agonist	Vasoconstriction (venous constriction stronger than arterial constriction) and increased systemic vascular resistance (SVR) Mean arterial pressure (MAP) augmented by increased SVR; minimal cardiac inotropic or chronotropic effects	Reflex bradycardia, hypertension, arrhythmias, decreased cardiac output (CO), visceral ischemia, extravasation necrosis	No change in flap blood flow with systemic administration (Banic et al., 1997) Decreased flap blood flow (Cordeiro et al., 1997) Decreased flap blood flow (Massey and Surgery, 2007)
Ephedrine	Chan et al. Chang et al. Fang et al. Harris et al. Monroe et al.	Synthetic sympathomimetic amine	Strong β-1 and α-1 adrenergic agonist Moderate β-2 agonist	Vasoconstriction 2/2 increased endogenous norepinephrine at post-synaptic receptors Increased inotropic/chronotropic effects due β activity Increased SVR due to α-1 receptor activity	Tachycardia, arrhythmias, splanchnic vasoconstriction	NA
Calcium chloride	Fang et al.	Inotropic and vasoactive agent	Ca/calmodulin- dependent kinase II (CaMKII) pathway	Increased calcium concentrations activate CaMKII pathway resulting in increased CO 2/2 ionotropic effects	Hypercalcemia, hypotension, arrhythmia, bradycardia, syncope	NA
Norepinephrine (Levophed)	Rose et al.	Synthetic and endogenous catecholamine	Strong α-1 adrenergic agonist Moderate β-1 agonist	Vasoconstriction (increased SVR) resulting in CO	Severe reflex bradycardia, visceral ischemia, hypertension	Increased blood flow (Eley et al., 2013)
Metaraminol	Rose et al.	Synthetic sympathomimetic amine	Strong α-1 agonist	Vasoconstriction (increased SVR) resulting in CO; indirectly releases endogenous norepinephrine	Bradycardia, hypertension, arrhythmia	NA
Dopamine (Intropin)	Monroe et al. (Monroe et al., 2010)	Synthetic and endogenous catecholamine	Moderate D1 agonist Dose dependent β-1 and α-1 agonist	Low dose - selective vasodilation (decreased SVR) Intermediate dose - increased stroke volume and CO primarily due to beta adrenergic activity High dose - vasoconstriction (increased SVR and CO) due to alpha activity	Tachycardia, hypotension, arrhythmias, polyuria, extravasation necrosis	No change in flap blood flow (Cordeiro et al., 1997)
Dobutamine (Dobutrex)	NA	Inotropic agent	Strong β-1 adrenergic agonist Moderate β-2 agonist	Vasodilation (decreased SVR) Increased CO due to inotropic and chronotropic effects	Tachycardia, arrhythmia, headache, nausea	Increased flap flow (Cordeiro et al., 1997) Increased flap flow (Eley et al., 2013) Increased pedicle blood flow (Scholz et al., 2009)
Epinephrine (Adrenalin)	NA	Synthetic and endogenous catecholamine	Strong β-1 and α-1 adrenergic agonist Moderate β-2 agonist	Low dose - increased CO due to inotropic/chronotropic effects (β activity) High dose -Increased SVR and CO due to α-1 receptor activity	Tachycardia, arrhythmias, angina, extravasation necrosis, splanchnic vasoconstriction, pulmonary edema	Increased flap flow (Massey and Surgery, 2007) Decreased flap flow (Eley et al., 2013)
Vasopressin	NA	Endogenous hormone *vasopressor-like effects occur during sepsis/shock	Specific vascular (V-1) and renal (V-2) receptors	Vasoconstriction due to increased intracellular calcium; increased MAP Decreases nitric oxide-mediated vasodilation	Myocardial ischemia, arrhythmias, hyponatremia, bronchospasm, skin necrosis	NA

During free flap harvest, it has been postulated that local catecholamines are released due to the activation of sympathetic fibers induced during tissue dissection (Banbury et al., 1999). Once the local supply of catecholamines is depleted, the acute hyperadrenergic phase is followed by a non-adrenergic phase with possible increased collateral blood flow, and then by an increased adrenergic phase due to loss of modulating autonomic input (Banbury et al., 1999; Godden et al., 2000; Lecoq et al., 2008; Raittinen et al., 2016). The exact onset of adrenergic hypersensitivity that occurs with sympathetic denervation is unclear but seems to occur in a delayed fashion ranging from 48 h to 2 weeks (Banbury et al., 1999). Regardless, it appears that denervated soft-tissue does not respond in the same manner as the rest of the body when exposed to vasopressors. In animal studies, Lecoq et al. and Cordeiro et al. both demonstrated increased microcirculation to flap tissue (cutaneous and musculocutaneous pedicled flaps, respectively) secondary to increased mean arterial pressure (MAP), while there was decreased flow in normal tissue (Cordeiro et al., 1997; Lecoq et al., 2008). Moreover, in the only animal study to specifically examine the effects of flap perfusion in a free flap model, Banic et al., demonstrated no adverse effects on pedicle blood flow or free flap microcirculation with the systemic use of phenylephrine (Banic et al., 1997). Thus, the use of vasopressors intraoperatively may actually increase flap perfusion due to improved overall MAP without significant deleterious effects from sympathectomy (Rizzoni et al., 2000; Goh et al., 2019).

### Physiology of the Autonomic Nervous System and Vasopressors

The autonomic nervous system, divided into parasympathetic and sympathetic components, regulates nearly every bodily function associated with homeostasis. Of particular interest to the microvascular surgeon are the adrenergic α- and β- receptors, which affect vascular tone and cardiovascular function. The role of α-1 agonists is to promote smooth muscle contraction, resulting in increased systemic vascular resistance and mean arterial pressure (MAP). α-2 agonists counteract this effect by causing smooth muscle relaxation. Of note, α-2 agonists also contribute to platelet aggregation through activation of α-2 receptors on platelets (Hoffman et al., 1982). β-1 agonists primarily enhance cardiac output due to positive inotropic and chronotropic effects, while β-2 agonists cause smooth muscle relaxation in the lungs (Miller and Pardo, 2011). Perioperatively, a host of endogenous and synthetic adrenergic agonists are available for use, each with varying degrees of effect on adrenergic receptors and associated physiologic and side effects (**Tables 2** and **3**) (Manaker and Parsons; Barrett et al., 2007; Miller and Pardo, 2011; Goh et al., 2019).

**Table 3:** Physiologic Effects of Common Vasopressors.

Drug	Common dosing range (µg/kg/min)	α-1	β-1	β-2	Vasopressin-1
Dobutamine	2–20	+	+++	++	0
Dopamine	2–20	++	+++	++	0
Epinephrine	0.01–0.15	+++	++	+	0
Norepinephrine	0.01–0.1	+++	++	++	0
Phenylephrine	10–20	+++	0	0	0
Vasopressin	0.01–0.07	0	0	0	+++
**Drug**	**Common dosing range (µg/kg/min)**	**Mean Arterial Pressure**	**Heart Rate**	**Cardiac Output**	**Systemic Vascular Resistance**
Dobutamine	2–20	+	+	+++	−
Dopamine	2–20	+	+	+++	+
Epinephrine	0.01–0.15	+	++	++	++
Norepinephrine	0.01–0.1	+++	−	−	+++
Phenylephrine	10–20	+++	0	0	+++

A variety of vasoconstrictive agents have been studied in animal models and used in human free tissue transfers, but the most commonly used vasopressors in the selected head and neck studies were ephedrine and phenylephrine. The prevalence rates in head and neck reconstruction was fairly common and range between 53.3% and 88.4%. Vasopressors are often required for patients undergoing general anesthesia in order to maintain MAP as volatile anesthetic agents can decrease SVR as well as blood loss, hypothermia, and insensible fluid losses. Phenylephrine is a commonly used α-1 adrenergic agonist with mixed results in animal models regarding flap perfusion (Banic et al., 1997; Cordeiro et al., 1997; Massey and Surgery, 2007). Ephedrine is an indirect sympathetic agonist with primarily strong β-1 and β-2 effects and weak α-1 stimulation. It is considered to be a suitable option in free tissue transfer due to lesser effects on peripheral vasoconstriction.

### Vasopressor Use in MFTT

While conflicting data exists, the majority of included studies indicate no increase rate of free flap failure with various vasopressor use (**Table 1**). Although, Chang et al. showed that the use of any vasopressor was a significant risk factor for arterial complication (Chang et al., 2017). However, this finding may be confounded by the underlying etiology of hypotension that resulted in the need for vasopressor use (Chang et al., 2017). It is also important to note that in this study arterial compromise did not significantly affect overall flap survival.

It has been postulated that a perforator flap may be more susceptible to intraoperative and postoperative complications with the use of vasopressors due to smaller caliber vessels. However, Harris et al. observed no significant association between the use of intraoperative vasopressors and postoperative complications specifically in free perforator flaps (p = 0.22) (Harris et al., 2012). In a prospective study of 24 patients undergoing head and neck resection and free flap reconstruction, Eley et al. observed that postoperative use of dobutamine and norepinephrine improved free flap skin blood flow (Eley et al., 2013). This increase in free flap perfusion with dobutamine has been observed by others and may be due to the β-adrenergic selectivity and isolated inotropic characteristics (Cordeiro et al., 1997; Suominen et al., 2004; Scholz et al., 2009). In a randomized controlled trial, Raittenen et al. randomized 25 patients undergoing radial forearm free flaps into three groups: dopamine, norepinephrine, and a control group with the goal vasopressor administration to maintain a MAP of 80 to 90 mmHg. The continuous partial pressure of oxygen and lactate to pyruvate ratio was monitored intraoperatively and for 72 h postoperatively *via* a subcutaneous catheter in the free flap. The authors found no difference in free flap failure rate, complication rate, or in the aforementioned clinical variables among the three groups (Raittinen et al., 2016). While small, this study represents the highest level of evidence available when interrogating the use of vasopressors and concludes that these agents can be safely used in MFTT.

Moreover, in a recent meta-analysis, Goh et al. observed no significant difference in total flap failure rate between vasopressor (71/3444) and non-vasopressor (25/349) groups who underwent head and neck reconstruction (2.1% vs 3.3%; OR = 0.91, p = 0.72) (Goh et al., 2019). A separate meta-analysis by Swanson et al. demonstrated similar findings and showed that intraoperative vasopressors had no effect on the incidence of flap failure (2.9% vs 3.6%; OR = 0.68, p = 0.48) or complication rates (16.8% vs 18.6%; OR = 0.92, p = 0.71) (Swanson et al., 2016).

### Intravenous Fluid and ERAS Implications

The regulation of regional blood flow and overall hemodynamics including intraoperative MAP is a critical factor to maintain microvascular flap perfusion and decrease postoperative complications and morbidity (Sigurdsson, 1995; Haughey et al., 2001; Ibrahim et al., 2014; Goh et al., 2019). Many patients undergoing head and neck reconstruction are at risk for intraoperative hypotension due to anesthetic agents, opioid analgesics, prolonged operative times, blood loss, insensible fluid loss, hypothermia, and other associated medical co-morbidities (Sigurdsson, 1995; Chan et al., 2013; Raittinen et al., 2016; Goh et al., 2019). The administration of intravenous fluids (IVF) is often the first line treatment for acute hypotension prior to the use of vasopressors. The use of IVF may combat acute changes in blood pressure, but excessive IVF administration during prolonged surgical cases can result in flap edema, decreased flap microcirculation, and flap complications (Haughey et al., 2001; Goh et al., 2019). Free flaps are likely predisposed to significant edema due to the absence of lymphatic drainage pathways and poor interstitial fluid reabsorption secondary to flap denervation (Sigurdsson, 1995; Goh et al., 2019). Moreover, excessive fluid resuscitation can lead to pulmonary edema and associated cardiopulmonary complications, which can also lead to subsequent deleterious flap and patient outcomes (Fang et al., 2018). Hand et al. observed that patients suffering perioperative complications received on average 525 mL more crystalloid than patients without complications (Hand et al., 2015). Similarly, Eskander et al. noted a 1.21 fold increased risk of wound-healing complications with each additional liter of crystalloid administered intraoperatively (Eskander et al., 2018). On the contrary, under-resuscitation can also result in increased free flap complications due to poor flap perfusion, worsened by further hypotension secondary to anesthetic agents (Sigurdsson, 1995). Thus, when faced with cardiovascular instability intraoperatively, anesthesiologists and microvascular surgeons must balance the use of vasoactive medications and fluid administration in an effort to improve free flap and patient outcomes.

Free tissue transfer for head and neck reconstruction often requires prolonged operative times and long hospitalizations (Won et al., 2019). ERAS protocols specific to head and neck surgery have been proposed and implemented to help reduce surgical complications and enhance recovery by utilizing a multimodal and multidisciplinary approach (Coyle et al., 2015; Dort et al., 2017). Goal-directed fluid replacement (equal weight in the immediate pre- and postoperative period) is one of several ERAS principles (Dort et al., 2017). The recent addition of stroke-volume variation to guide IVF administration has been shown to reduce length of stay and medical complications in head and neck patients (Abdel-Galil et al., 2010). Surgical teams must be vigilant in monitoring total IVF replacement in the postoperative period while also accounting for the volume of intraoperative fluid resuscitation in order to reduce flap edema, complications, and other perioperative morbidities. Thus, the use of intraoperative vasopressors to regulate systemic perfusion pressure may actually be a beneficial alternative to fluid administration to improve flap perfusion (Wax and Azzi, 2018).

### Challenges With Assessing Vasopressor Use

As discussed, much of the literature available assessing vasopressor use has been limited to animal studies, other anatomic sites, or retrospective reviews. The lack of quality prospective data to draw from is a challenging issue. Currently, free flap success rates remain very high across the world, exceeding 95% at high volume centers (Densky et al., 2019). As such, many studies are under-powered and identifying statistically significant variables is not possible without extremely large patient volumes from multiple centers or across a time frame that introduces significant variations in clinical protocol. This type of data collection would certainly open the window for confounding variables and errors in data collection and analysis. Thus, there is no reasonable manner in which to perform a randomized, prospective trial in a safe, financially feasible, and ethical fashion. Lastly, patients undergoing surgery for advanced head and neck cancer frequently have significant medical comorbidities, which certainly play a role in the need for pressor support during general anesthesia. These comorbidities are difficult to control for in a meta-analysis due to inadequate powering.

### Clinical Experience With Vasopressor Use

Anecdotally, we have seen success with the use of vasopressor agents intraoperatively at our institution as we have recently instituted improved ERAS protocols. Combined with the use of neuromuscular blockage to reduce systemic anesthesia requirements, a low-dose pressor drip throughout the case can help stabilize MAP and has been subjectively improved flap perfusion after anastomosis. Our anesthesiologists prefer the use of a low-dose phenylephrine drip when necessary to counter anesthesia-related reduction of SVR. In our experience, when we encounter issues related to flap perfusion with the use of vasopressor agents, it is related to bolus administration and typically occurs immediately prior to or just after flap re-perfusion. It is possible that wide fluctuations in MAP related to bolus administration of vasopressors are detrimental to the peripheral blood flow needed to perfuse the MFTT. We fear that bolus-style administration is most worrisome when performed around the time of flap re-perfusion as vascular compromise at this critical juncture can have long-lasting negative effects for the MFTT outcome. For this reason, we prefer a continuously-infused, low-dose agent, such as phenylephrine, when vasopressor administration is required. Phenylephrine remains the first-choice agent in these common settings at our institution.

Along with this approach, we stress the importance of clear communication with the anesthesia team to coordinate vasopressor timing, dosage, and agent. Of note, although a survey in 2015 showed that 70% of microsurgeons do not permit the use of vasopressors in non-emergent settings (Motakef et al., 2015; Chang et al., 2017), the retrospective studies reviewed here showed vasopressor use in 53.3% to 88.4% of surgeries (Monroe et al., 2010; Monroe et al., 2011; Harris et al., 2012; Chan et al., 2013; Rose et al., 2016; Chang et al., 2017; Fang et al., 2018; Farquhar et al., 2018). This high prevalence rate suggests that vasopressor agents are administered more frequently than surgeons are aware of, further emphasizing the importance of adequate communication.

As the majority of results highlighted in **Table 1** suggest, the use of intraoperative vasopressors is not associated with worse free flap outcomes. This is seen regardless of agent choice and administration timing during the case. However, a thoughtful methodology and communication between teams must be implemented. The judicious and appropriate use of intraoperative vasoactive agents can even be beneficial when utilizing ERAS protocols which are becoming commonplace at high volume centers. Postoperative free flap complications including arterial complications are often multifactorial in nature and can be confounded by associated medical co-morbidities making quality evaluation of these issues difficult (Chang et al., 2017).

## Conclusion

Based on the results of the highlighted studies and recent meta-analyses, the use of intraoperative vasopressors appears to be safe in free tissue transfer for head and neck reconstruction. It is imperative to maintain open communication between microvascular surgeons and anesthesiologists in order to maintain a balance between the use of IVFs and vasopressors in the perioperative period. Prospective studies are warranted, taking advantage of ERAS protocols in place, to further examine the safety of intraoperative vasopressors on postoperative free flap outcomes and to establish evidence-based guidelines regarding the ideal type, dose, and timing of intraoperative vasopressor.

## Author Contributions

AN and NS contributed to the design and implementation of the project. AN, TF, ML, and NS performed the primary the literature review and data analysis. AN, ML, and NS wrote the manuscript after discussion, contributions, and edits from all authors (including AT, AA, EO, SK, MO).

## Conflict of Interest

The authors declare that the research was conducted in the absence of any commercial or financial relationships that could be construed as a potential conflict of interest.

The handling editor declared a shared affiliation, though no other collaboration, with the authors.
